# A controlled-release oral opioid supports *S*. *aureus* survival in injection drug preparation equipment and may increase bacteremia and endocarditis risk

**DOI:** 10.1371/journal.pone.0219777

**Published:** 2019-08-09

**Authors:** Katherine J. Kasper, Iswarya Manoharan, Brian Hallam, Charlotte E. Coleman, Sharon L. Koivu, Matthew A. Weir, John K. McCormick, Michael S. Silverman

**Affiliations:** 1 Department of Microbiology and Immunology, Western University, London, Canada; 2 Department of Medicine, Western University, London, Canada; 3 Department of Epidemiology and Biostatistics, Western University, London, Canada; 4 Department of Family Medicine, Western University, London, Canada; 5 Lawson Health Research Institute, London, Canada; 6 Division of Infectious Diseases, Western University, London, Canada; Laurentian, CANADA

## Abstract

**Background:**

Injection drug use-associated endocarditis (IDUaIE) incidence in Ontario has recently been associated with hydromorphone prescribing rates. *Staphylococcus aureus* causes the majority of cases of IDUaIE in Ontario and across North America. Hydromorphone controlled-release (Hydromorphone-CR) requires a complex technique for injection and therefore provides multiple opportunities for contamination. Hydromorphone-CR contains several excipients, which could enhance staphylococcal survival and increase risk of contaminating the injectate.

**Methods:**

Used injection drug preparation equipment (cookers/filters) was collected from persons who inject drugs (PWID), rinsed with water, and plated on Mannitol salt agar. Bacterial isolates from bacteremic PWID were used to assess the survival of *S*. *aureus* and *Streptococcus pyogenes* on cookers/filters with Hydromorphone-CR, hydromorphone immediate-release (Hydromorphone-IR) or oxycodone controlled-release (Oxycodone-CR). The solutions spiked with *S*. *aureus* were heated and the remaining viable bacteria enumerated.

**Results:**

*S*. *aureus* was detected in 12/87 (14%, 95%CI 8–23%) cookers/filters samples used for injection of Hydromorphone-CR. Hydromorphone-CR was the only opioid associated with greater survival of methicillin-sensitive *S*. *aureus* (MSSA) and methicillin-resistant *S*. *aureus* (MRSA) on cookers/filters when compared to sterile water vehicle control. There was a ~2 log reduction in the number of *S*. *aureus* that survived when cookers/filters were heated.

**Conclusion:**

14% of all cookers/filters used in the preparation of Hydromorphone-CR were contaminated with *S*. *aureus*. Hydromorphone-CR prolongs the survival of MRSA and MSSA in cookers/filters. Heating cookers/filters may be a harm-reduction strategy.

## Introduction

Infective endocarditis is a potential complication of injection drug use that is associated with substantial morbidity and mortality [1]. London, Ontario has a particularly high incidence of Injection Drug Use associated Endocarditis (IDUaIE) with 55% of all first episodes of Infective Endocarditis (IE) being IDUaIE [2]. This is in contrast to recent US surveys which have demonstrated that IDUaIE has been increasing, but still remains much less common with IDUaIE making up 8–13% of all IE cases [3,4]. Studies in many centers have demonstrated that *S*. *aureus* causes between 66–73% of all cases of endocarditis in PWID [5–8]. In London, Ontario, *S*. *aureus* is by far the predominant organism with 77.2% of first episode cases of IDUaIE being caused by this organism (113/202 (55.9%) methicillin-sensitive *S*. *aureus* [MSSA] and 43/102 (21.3%) methicillin-resistant *S*. *aureus* [MRSA])[2]. PWID are frequently noted to have nasal colonization with *S*. *aureus* [9]. The mechanism of staphylococci contaminating the injection process and thus gaining access to the bloodstream is under-investigated.

The opioid most commonly used in our PWID population is hydromorphone controlled-release (Hydromorphone-CR) (marketed in over 15 countries on 5 continents with several brand names [Hydromorph Contin] or Palladone SR [Purdue Pharma, Stamford, Connecticut] and as Jurnista, [Janssen-Cilag Pty Ltd, Macquarie Park NSW, Australia]). This opioid is extensively prescribed in Canada, particularly in Ontario, and due to diversion, it is widely available for use by local PWIDs [10]. IDUaIE has been increasing in Ontario and its incidence is closely associated with the Hydromorphone prescription rates [11]. Hydromorphone-CR contains several carbohydrates, iron oxide, and gelatin (a protein) as drug excipients, which individually or together may enhance bacterial survival [12].

**Fig 1:**
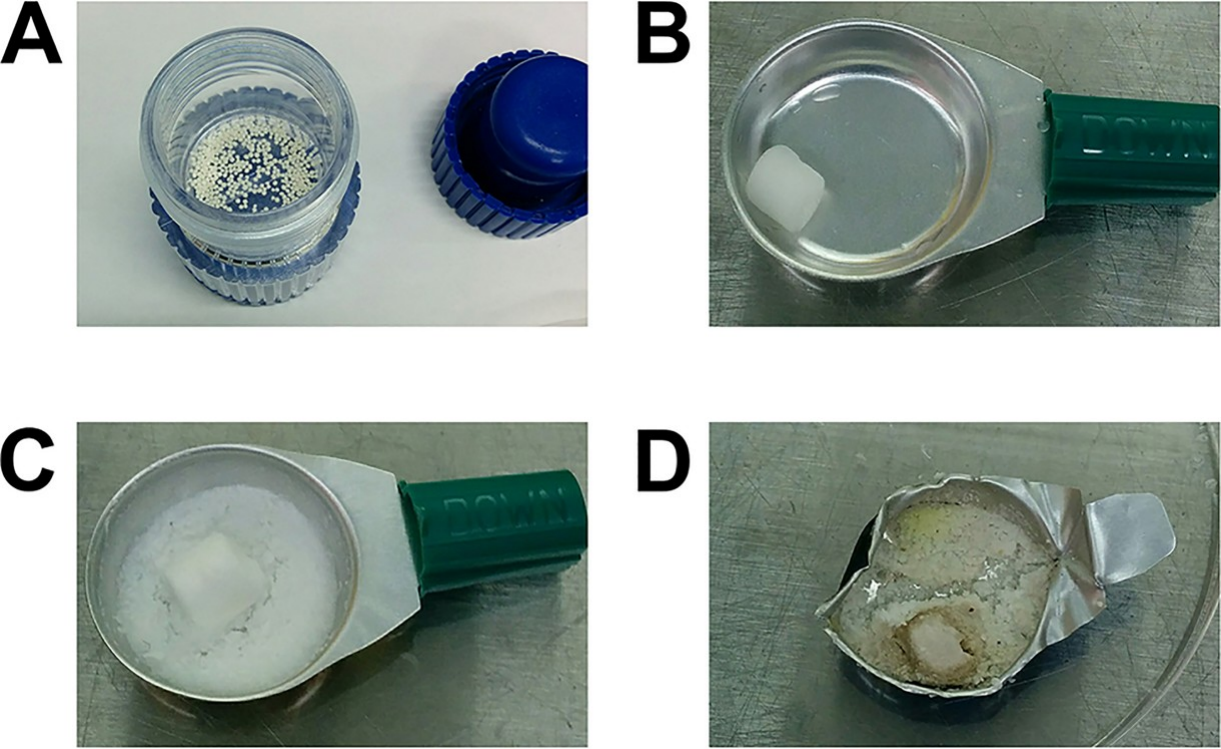
Opiates and injection equipment commonly used by PWID. (A) Pill crusher with uncrushed hydromorphone controlled-release (Hydromorphone-CR) beads (removed from capsule). (B) Unused injection drug preparation equipment consisting of metal cooker and filter. This style of cooker with compressed cotton filter is distributed as part of a provincial harm-reduction initiative from the needle exchange along with clean needles, syringes and water. All cookers collected in the used equipment collection program have been in this style of cooker. (C) Hydromorphone-CR-containing sterile cooker and filter after a single wash in controlled laboratory environment. The large volume of remaining material available for repeat “washes” to get further opioid is visible. (D) Used cookers after three washes (donated by a person who injects drugs) with significant residual Hydromorphone-CR left in cooker. The cooker is often folded to retain residual opiate for later use and a “repeat wash”.

Hydromorphone-CR is unusual amongst prescription opioids in that it comes as a capsule containing multiple time-released beads, unlike hydromorphone immediate-release (Hydromorphone-IR, Dilaudid [Purdue Pharma, Stamford CT]), or oxycodone controlled-release (Oxycodone-CR, Oxycontin [Purdue Pharma, Stamford CT]) which are tablets. Hydromorphone-CR beads require crushing and dissolution in water to enable injection (Fig 1) [13–15]. The equipment used by PWID for this process typically consists of cookers (small pans used to mix and then aspirate the preparation) and filters to prevent aspiration of large particles into the syringe (Fig 1). The drug excipients of Hydromorphone-CR contribute to the low water solubility of the drug, and therefore require PWID to perform multiple “washes” (addition of further water and re-aspiration of residual drug) as well as reuse of a filter in order to avoid “wasting” any undissolved opioid [13]. Due to the high cost (street value approximately $60 CDN or $45USD/capsule), the residual Hydromorphone-CR left in the filter is often stored for reuse hours to days later. This process requires extensive manipulation and allows multiple opportunities for contamination.

We hypothesized that the complex methods needed for preparation of Hydromorphone-CR for injection would lead to frequent handling and contamination of cookers/filters with *S*. *aureus*. Furthermore, we hypothesized that the excipients within Hydromorphone-CR that allow it to be a controlled-release preparation would also increase *S*. *aureus* survival and by extension, increase contamination of the injectate (the fluid in the syringe which is injected intravenously). Lastly, we hypothesized that the act of “cooking the wash” (holding a cigarette lighter under the cooker until the solution starts bubbling [<10 seconds]) would impair the ability of *S*. *aureus* to survive on used cookers/filters and may thus protect patients who inject from *S*. *aureus* bacteremia.

## Methods

### *S. aureus* survival in drugs over time

Drugs to be tested were crushed in a pill crusher until the powder was homogeneous. Hydromorphone-IR (8 mg) and Oxycodone-CR [80 mg] are both tablets and so the entire tablet was crushed (see S1 Table). For Hydromorphone-CR, which is in a capsule with controlled-release beads inside, the capsule was removed and only the controlled-release beads were crushed (as per routine practice of PWID). The crushed drug powder was transferred to a 15 mL screw cap tube. *S*. *aureus* were prepared to be 10^4^ cells/ml (See Supporting Information 1) and 5 ml of the bacterial preparation was added to each tube and was thoroughly mixed. Experimental samples were incubated at ambient room temperature. At each time point, 100 μL of the sample was neat plated, serially diluted, and drop plated to determine bacterial survival at that time point. Opioids tested included Hydromorphone-CR, Hydromorphone-IR, and Oxycodone-CR (S1 Table). Drug dosages chosen were those most commonly injected by PWIDs. Bacterial persistence was assessed as the number of live bacteria recovered at each time point over 96 hours.

### Bacterial survival on cookers/filters

Strains of *S*. *aureus* (MSSA and MRSA) and *Streptococcus pyogenes* (*S*. *pyogenes*) isolated from bacteremic local PWID were prepared into an inoculation concentration of 10^4^−10^5^/mL (S1 Methods and S2 Table). *S*. *pyogenes* was included to ascertain if there would be increased survival with other Gram-positive organisms apart from *S*. *aureus* in solutions of Hydromorphone-CR. The methods for preparation of the S.aureus and S. pyogenes solutions are described in Supporting Information 1.

In order to mimic the technique that PWID use to prepare Hydromorphone-CR for injection, Hydromorphone-CR (24mg) beads were removed from the capsule, crushed in a generic pill crusher (Fig 1) until homogeneous, and the drug powder was transferred to a sterile aluminum cooker where it was re-suspended in 2 mL of bacterial preparation [13–15]. Similar to the techniques outlined by PWID, the drug preparation was allowed to soak in the cooker for approximately 5 minutes with occasional stirring with a needle. After the 5-minute soak, the drugs were drawn up into a syringe through the sterile compressed cotton filter that was provided with the sterile cooker. All experiments were performed in a certified biological safety cabinet with unused needles/syringes and injection drug preparation equipment (i.e. sterile water, cookers, and filters; Sterifilt^TM^ Apothicom, Paris, Fr) that were distributed as part of the provincial harm reduction program, and thus commonly used by local PWID. Samples were serially diluted and plated neat onto appropriate agar and the number of bacteria were enumerated. Subsequent washes were performed by adding 1 mL of sterile water and allowing the water to soak the drug and filter for 5 minutes before removing the liquid with a 25G needle and syringe through the filter. Between samplings, the cookers were stored at ambient room temperature in individual covered sterile petri dishes. If the drug was “cooked”, it was done during the 5-minute soak by holding a commercial BIC INC (North York, Ontario) butane cigarette lighter under the cooker until bubbles formed in the cooker (<10 seconds) (performed at 251 meters above sea level). All experiments were performed in triplicate.

### Assessment of cookers/filters bacterial burden in PWID community samples

Between March 2017 and March 2018, we collected 87 used cookers/filters from active PWID who used Hydromorphone-CR and were attending a local needle exchange clinic in London, Ontario. Donors were provided with a $5 coffee gift card in return for their used cookers/filters. Verbal consent for participation was obtained. All donations of cookers/filters were anonymous with the only information obtained being the type of drug used in the cookers/filters.

On receipt, the equipment was stored at 4°C prior to transport to the laboratory where 1 mL sterile water was added to the cooker and then aspirated to simulate an additional wash of the cookers/filters. If the sample was turned in with a filter included, the sample was drawn through the filter with a 25G needle. If there was no filter included, the sample was drawn up with a pipet. The wash was subsequently plated on mannitol salt agar and incubated for 24 hours at 37°C. Typical mannitol fermenting colonies identified on mannitol salt agar were Gram-stained. If Gram-positive cocci were identified, they were then assessed for production of coagulase to determine whether the strains collected were *S*. *aureus*. The resistance status of *S*. *aureus* was determined based on zones of inhibition adapted in part from CLSI document M100-S21 [16,17].

The study protocol was approved by the Lawson Health Research Institute institutional review board.

### Statistical methods

All experiments were performed in triplicate. Pearsons test was used to compare the proportion of cookers/filters which were used for Hydromorphone-CR or Hydromorphone-IR injection which were contaminated with *S*.*aureus*. Means are displayed with error bars representing 95% confidence limits. Comparisons of log10 resultant bacterial yield were compared using a one-way ANOVA with Tukey’s post-hoc test.

## Results

### Persistence of bacteria in drugs over time

**Fig 2:**
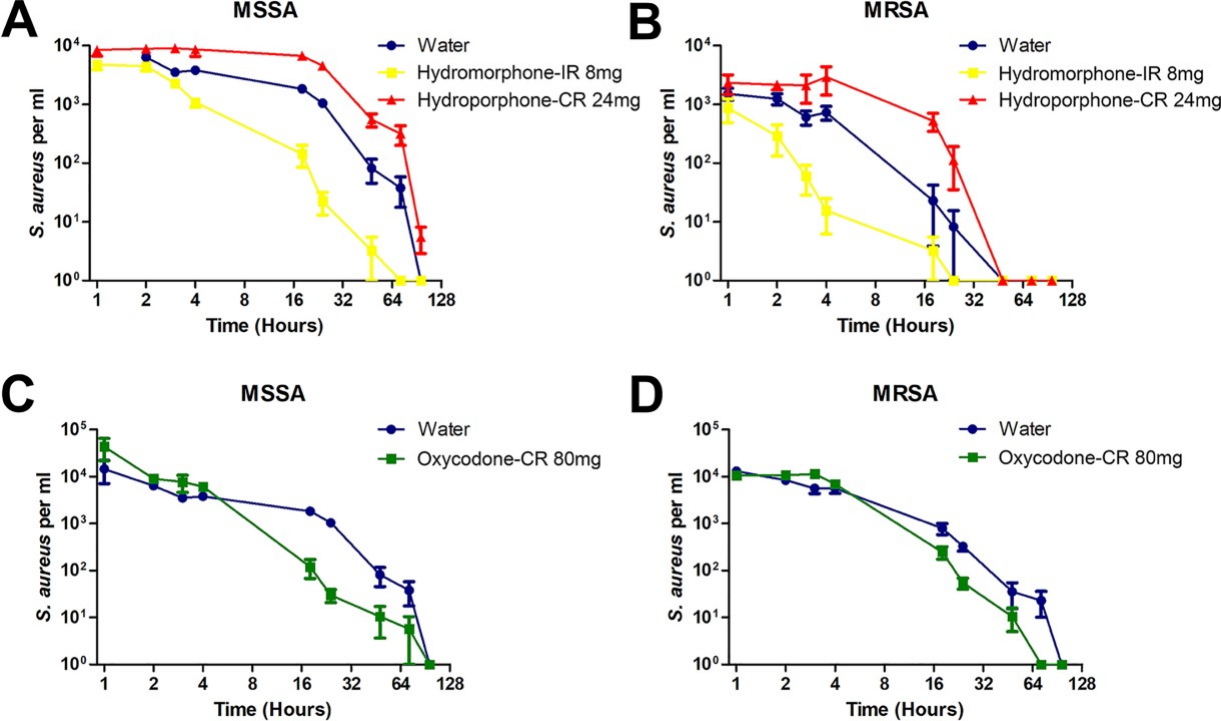
Persistence of *S*. *aureus* over time in immediate-release or controlled-release opioid solutions compared to sterile water control. (A-B) Clinical strains of *S*. *aureus*, both MSSA and MRSA, persisted longer in Hydromorphone-CR solution compared to both hydromorphone immediate-release (Hydromorphone-IR) solution or sterile water control. (C-D) Both MSSA and MRSA persisted longer in sterile water control when compared to oxycodone controlled-release (Oxycodone-CR) solution.

*S*. *aureus*, both MRSA and MSSA, survived in greater numbers in the presence of Hydromorphone-CR as opposed to Hydromorphone-IR or sterile water. Interestingly, *S*. *aureus* survived longer in the sterile water vehicle control than in Hydromorphone-IR (Fig 2A and 2B). Similar to Hydromorphone-IR, we observed that *S*. *aureus* had reduced survival duration when Oxycodone-CR was placed in the Cookers/Filters when compared to sterile water (Fig 2C and 2D).

### Bacterial survival on cookers/filters

**Fig 3:**
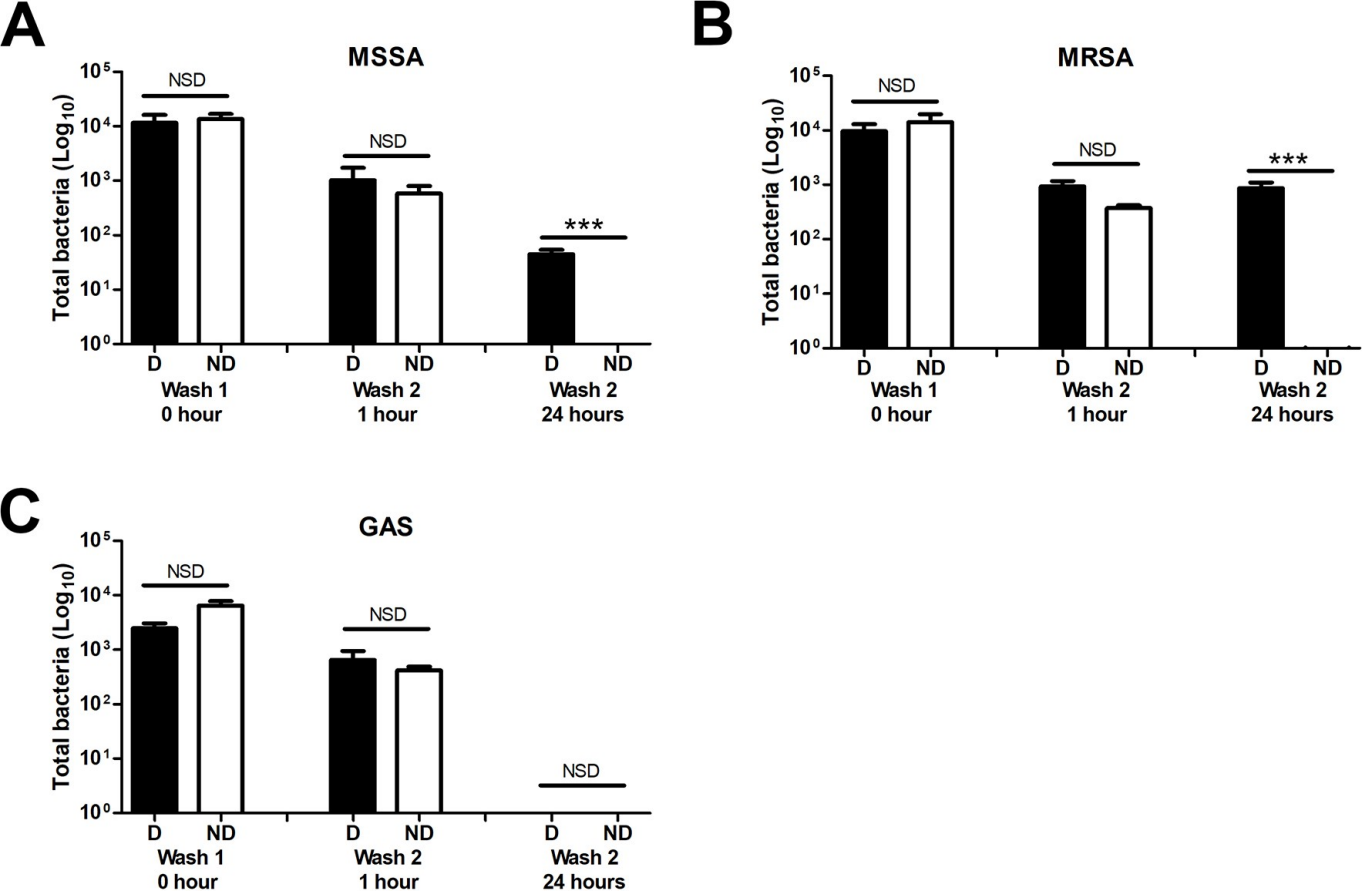
Bacterial survival of clinical strains of MSSA, MRSA, and *S*. *pyogenes* on unused Cookers/Filters containing Hydromorphone-CR solution or sterile water control. All Cookers/Filters underwent an initial wash with bacteria-spiked water for 5 minutes and were subsequently left dry at room temperature before a second five minute wash was performed at either 1 hour or 24 hours. Both (A) MSSA and (B) MRSA survived significantly better in Hydromorphone-CR than in the water vehicle control when the second wash happens after 24 hours. (C) *S*. *pyogenes* was not isolated from the drug equipment after 24 hours. Significance assessed by one way Anova with Tukey’s correction for multiple comparisons. ***P<0.001, NSD indicates no statistically significant difference. D = Drug, (Hydromorphone-CR), ND = No drug (sterile water vehicle control).

We found that Hydromorphone-CR was associated with greater persistence of MSSA and MRSA (but not *S*. *pyogenes*) in solutions of the drug, when the wash was performed at 24 hours compared to sterile water vehicle control (Fig 3) (p<0.001). Hydromorphone-CR did not prolong survival of *S*. *pyogenes* at 24 hours and in fact, little to no bacteria remained in the drug solution by that time.

### Contamination by *S. aureus* is reduced by “cooking” cookers/filters

**Fig 4:**
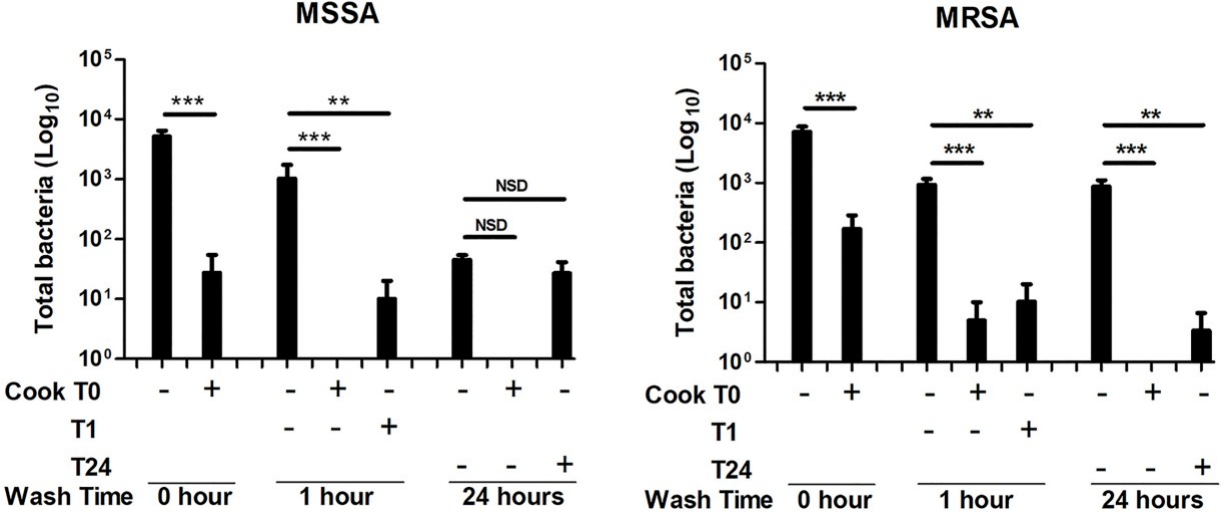
Cigarette lighter heating of *S*. *aureus*-"spiked" Hydromorphone-CR prepared in cookers/filters decreases the bacterial burden in the injected drug solution. *S*. *aureus*-spiked samples in Hydromorphone-CR-containing Cookers/Filters were heated with a cigarette lighter (holding the flame under the cooker until bubbling) during either the initial wash or at the subsequent 1-hour or 24-hour second wash. Heating reduced *S*. *aureus* bacterial recovery by approximately 2 log10 when performed immediately after bacterial contamination, but delayed heating was less effective. Significance was assessed by one way Anova with Tukey’s correction for multiple comparisons. **P<0.01, ***P<0.001, NSD indicates no statistically significant difference.

We found that when the solutions of Hydromorphone-CR or Hydromorphone-IR spiked with *S*. *aureus* were “cooked” [heated in the cooker with a cigarette lighter until bubbling (<10sec)], there was a ~2 log10 reduction in the number of viable bacteria when performed immediately after bacterial contamination, but delayed heating was less effective (Fig 4).

### Used cookers/filters contamination with *S. aureus*

*S*. *aureus* was detected in 12/87 (14%, 95%CI 8–23%) cooker/filter samples that had been used for injection of Hydromorphone-CR (6 were MRSA, 5 were MSSA and 1 was Borderline Oxacillin Resistant *S*. *aureus* (BORSA) [18].

## Discussion

Our data demonstrates that 14% of cookers/filters that had been used by PWID for injection of Hydromorphone-CR were contaminated with *S*. *aureus*. This was particularly remarkable as the equipment was often stored at room temperature for several days before transfer to the laboratory, limiting the sensitivity of testing and so, the true prevalence of *S*. *aureus* contaminated cookers/filters may be even higher. We used the same technique to sample the cookers/filters that PWID would use for a “wash” and therefore the *S*. *aureus* which we cultured would have been injected intravenously during routine use.

We also demonstrated that Hydromorphone-CR could prolong *S*. *aureus* survival *in vitro* in contrast to Hydromorphone-IR and Oxycodone-CR which failed to do so. As this effect was not seen with Hydromorphone-IR, we do not believe that it is a property of the opioid itself, but rather the drug excipients present that make it a controlled-release preparation. These excipients include several carbohydrates (ethyl cellulose, hydroxypropyl methylcellulose, microcrystalline cellulose), a protein (gelatin) and iron oxide which all may enhance *S*. *aureus* survival [12]. In contrast Hydromorphone-IR contains only one carbohydrate (lactose) and does not contain iron or gelatin and thus may not have this effect [19]. It is notable that the drug excipients in Hydromorphone-CR and Oxycodone-CR are quite different [19,20]. The mechanism for making Oxycodone-CR a delayed release preparation involves use of ammonio-methacrylate copolymer (water-insoluble polymer) and stearyl alcohol (water-insoluble wax), and thus would not be expected to support *S*. *aureus* survival [21]. Further studies are ongoing to identify which specific excipients within Hydromorphone-CR may be producing this effect. The complex technique with frequent handling required to prepare Hydromorphone-CR for injection, as well as the combination of its excipients which support bacterial survival, may help to explain the high incidence of IDUaIE in London, Ontario with its known predilection for Hydromorphone-CR injection. This data also helps to explain the recent observation that rates of PWIDaIE have been rising across Ontario with a time course which is temporally associated with increasing prescriptions for hydromorphone [11].

The marked reduction in bacterial load associated with heating (“cooking”) cookers/filters prior to aspiration indicates that this may be a simple but beneficial harm reduction approach. The fact that this occurred with heating for <10 seconds using a routine cigarette lighter suggests that this approach is feasible for PWID to perform. Our data also showed that heating cookers/filters was less effective when it was delayed to 24 hours as opposed to with each wash, particularly in the solutions with MSSA (Fig 4). We believe that this may be due to the bacteria becoming deeply embedded in the filter and left over drug material within the cookers/filters over time and thereby resulting in poor heat transfer. This suggests that the most effective method would be to heat cookers/filters during every wash.

In addition to endocarditis, bacterial complications of injection drug use can involve skin and soft tissue, musculoskeletal, pulmonary, neurological as well as endovascular infections [22]. Prior studies have shown that cleaning the skin prior to injection may help to reduce the risk of developing local skin infections [23]. Our data demonstrates frequent *S*. *aureus* contamination of cookers/filters and the resulting injectate, suggesting that skin cleaning alone is unlikely to be sufficient and heating of the injectate may be an important public health intervention.

We have recently shown that the excipients within Hydromorphone-CR also preserve HIV viability and that sharing of cookers/filters can promote HIV transmission [14,15]. Heating cookers/filters was shown to reduce HIV survival. Heating cookers/filters containing Hydromorphone-CR or Hydromorphone-IR does not significantly change the amount of hydromorphone released into the injectate and so should not increase the risk of overdose [14,15]. We are presently conducting a community campaign to “cook your wash” in an effort to reduce the high local incidence of HIV and infective endocarditis [24]. Results of this campaign on endocarditis incidence will be presented subsequently.

Increasing hospitalization of PWID in the United States, especially for infection, have sparked interest in improving preventions of infections in PWID [25]. Recent CDC data demonstrated that PWID were approximately 16.3 times more likely to develop invasive MRSA infections than non-injection drug users and injection drug use accounted for an increasing proportion of invasive MRSA cases, rising from 4.1% to 9.2% between 2011–2016 [26].

We observed that 14% of used cookers/filters (but not all) were contaminated with *S*.*aureus*. In order for the cookers/filters to become contaminated with *S*.*aureus* the PWID who was handling and preparing the drug for injection would have to be colonized or infected with the organism. A large general population survey found that approximately 32% of the US population was colonized with *S*.*aureus* [27]. A survey of PWID in New York found that approximately 20% were nasally colonized with *S*.*aureus* [28]. A further 14% were negative on nasal swab culture but had a skin infection within the past 30 days which was suggestive of recent staphylococcal infection. Therefore our data would suggest that a large percentage of PWID who are colonized or infected with *S*. *aureus* contaminate their cookers/filters during Hydromorphone-CR injection preparation. This likely is related to the multiple complex steps involved in the preparation process and the storage for repeated use.

Although 14% of cookers or filters became contaminated when preparing Hydromorphone-CR, we do not believe that it is due to the cookers/filters themselves as none of the unused equipment which we collected from the harm reduction site was contaminated. Therefore we do not feel that distribution of this equipment should cease. As noted in the introduction, the high street value of Hydromorphone-CR and large amount remaining after an initial wash is strongly motivating for storage and reuse. In the absence of commercial cookers and filters patients tend to use other household equipment such as spoons and cigarette filters for preparation and storage. We suspect that this is a more dangerous alternative but further study is warranted.

It has been proposed that some opiates can have greater infectious risks because they require a larger gage needle to inject due to poor water solubility. We do not think it is likely that this is leading to a higher rate of infectious complications in our setting as London Ontario has had an exceptionally large needle/syringe distribution program for several years with over 3 million needles given out in 2017 in a population of ~400,000. This is the largest needle distribution program per capita in Canada and one of the largest in the world [29]. Therefore, almost all PWID access their needles from the common Provincial source which provides a choice of needle gage of 27, 28 or 29 with 27 most commonly used. All PWID are provided filters along with their cookers and needles/syringes to enable the solution to be drawn up into the syringe. Therefore almost all of our local PWID use very small gauge needles.

Prescription opioids may also predispose to bacterial infections via immune-compromising effects [30–33]. An increased incidence of invasive pneumococcal disease has been described in patients prescribed opioid analgesics, particularly those taking long acting, potent opioids [34]. Unlike morphine and fentanyl which are felt to have immune-compromising effects *in vitro* and in animal models, hydromorphone and oxycodone are not felt to have this same effect [32–34]. That said, recent studies showed that both immune-compromising and non-immune compromising agents (including hydromorphone) demonstrated an association with infection [30,34].

We noted that Hydromorphone-CR increased survival of *MSSA* and *MRSA* but not *S*. *pyogenes*. This likely is due to the fact that *S*. *pyogenes* is a much more fastidious bacterium than *S*. *aureus*. Unlike *S*. *aureus*, *S*. *pyogenes* lacks the enzymes for the citric acid cycle and oxidative-cytochromes for the electron transport chain, and is thus completely reliant on the fermentation of sugars for growth and energy production [35,36]. Additionally, *S*. *pyogenes* is considered a multiple amino acid auxotroph and requires nearly all amino acids to be present in the growth media. In fact when compared to *S*.*aureus* growth media, *S*. *pyogenes* requires 12 additional amino acids to be added to the minimal growth media to support its growth [35–37]. Nevertheless S. pyogenes is an important pathogen in PWID with multiple outbreaks of disease associated with this organism, including in London, Ontario [38]. We suspect that crowding within homeless shelters, frequent soft tissue injury, as well as pre-injection practices (such as licking of the needle) may lead to these infections rather than contamination of the injectate.

### Study limitations

We did not have access to cookers/filters which had been used to prepare Hydromorphone-IR. This is related to the high solubility of Hydromorphone-IR and thus PWID did not tend to preserve their cookers/filters for subsequent “washes”[14,15]. This presumably led to less bacterial contamination from prolonged storage and possibly lower infection risk. We also did not have any cookers/filters which had been used to prepare Oxycodone-CR as this product was removed from the Canadian market due to its high addiction risk although it is still widely used in the USA and Europe. We do not have donor demographic data to correlate with cookers/filters contamination as used cookers/filters donation occurred in an anonymous setting with the only information attached being drug used in the cookers/filters prior to donation. Nasal colonization rates with *S*.*aureus* were previously found to be lower in PWID of African descent [28]. A general population survey also found lower *S*. *aureus* nasal colonization prevalence in persons of African race or Mexican descent, but higher MRSA colonization rates in persons 65 years of age or older, women, persons with diabetes, and those who were in long-term care in the past year [27]. Therefore, the frequency of cooker/filter contamination may be different in different demographic groups.

Although “cooking the wash” reduced the number of bacteria in the injectate some bacteria survived the process. Nevertheless, it has been known for many years from studies of experimental endocarditis in animals that the production of a bacterial vegetation by intravenous injection is inoculum dependent [39, 40]. This has been the principal behind antibiotic prophylaxis of infectious endocarditis. We therefore believe that the approximately 2 log decrease in the bacterial load injected associated with “cooking your wash” may lead to a reduction in endocarditis incidence.

## Conclusion

14% of all cookers/filters that had been used for the preparation of Hydromorphone-CR (but not Hydromorphone-IR) were contaminated with *S*. *aureus*. *In vitro* survival conditions showed that Hydromorphone-CR, (but not Hydromorphone-IR or Oxycodone-CR) prolonged the survival of MRSA and MSSA. This enhanced bacterial survival is likely due to the drug excipients used to make Hydromorphone-CR a controlled-release preparation. Heating cookers/filters with a cigarette lighter prior to each injection may be an effective harm reduction technique that could help prevent bacterial contamination of the injectate, and potentially reduce bacterial complications of injection drug use.

## Supporting information

S1 Methods Preparation of staphylococcal and streptococcal solutions. (DOCX)

S1 Table The opioids used in this study. (DOCX)

S2 Table Clinical bacterial isolates from active PWID that were used in this study. (DOCX)

S3 Table Primary data. (XLSX)
