# Correction: A controlled-release oral opioid supports *S*. *aureus* survival in injection drug preparation equipment and may increase bacteremia and endocarditis risk

**DOI:** 10.1371/journal.pone.0223079

**Published:** 2019-09-19

**Authors:** Katherine J. Kasper, Iswarya Manoharan, Brian Hallam, Charlotte E. Coleman, Sharon L. Koivu, Matthew A. Weir, Johan Delport, John K. McCormick, Michael S. Silverman

Johan Delport is not included in the author byline. Johan Delport should be listed as the seventh author and affiliated with Department of Medical Microbiology, London Health Sciences Center, London, Canada. The contributions of this author are as follows: Methodology and Validation.
