# Supplementary material for: A controlled-release oral opioid supports S. aureus survival in injection drug preparation equipment and may increase bacteremia and endocarditis risk
Source: PLoS One. 2019 Aug 9;14(8):e0219777. doi: 10.1371/journal.pone.0219777 (PMC6688832; doi:10.1371/journal.pone.0219777)
Supplement: S1 Methods — (DOCX) [file pone.0219777.s001.docx]

S1Methods

Preparation of Staphylococcal and Streptococcal solutions

*S. aureus* was prepared by growing the bacteria from -80°C stocks in brain heart infusion media (BHI) at 37°C with shaking overnight before being sub-cultured (at 1%) into 50 mL of 37°C pre-heated BHI in a flask to grow for 3 hours (early logarithmic phase). Staphylococci were then pelleted, washed in 1🞨 sterile Hank’s Balanced Saline Solution and re-suspended to OD_600_=1±0.1, which is equivalent to ~10^8^ bacteria per mL. Ten-fold serial dilution in 10 mL volumes of Hank’s Balanced Saline Solution was performed to 10^-4^ and the final 10-fold serial dilution to the working solution was made into water. *S. pyogenes* was grown from -80°C cultures in Todd-Hewitt media supplemented with 1% yeast extract (THY) at 37°C in a screw cap tube without shaking before being sub-cultured (at 1%) into a 100 mL screw cap bottle of pre-heated THY to grow for 3 hours (early logarithmic phase)) at 37°C without shaking. *S. pyogenes* was then prepared in the same fashion as *S. aureus*
