# Supplementary material for: A controlled-release oral opioid supports S. aureus survival in injection drug preparation equipment and may increase bacteremia and endocarditis risk
Source: PLoS One. 2019 Aug 9;14(8):e0219777. doi: 10.1371/journal.pone.0219777 (PMC6688832; doi:10.1371/journal.pone.0219777)
Supplement: S1 Table — (DOCX) [file pone.0219777.s002.docx]

S1 Table. The opioids used in this study

| Drug | Abbreviation | Dose (mg) | Format | DIN/CN | Appearance |
| --- | --- | --- | --- | --- | --- |
| Hydromorph Contin  (Purdue Pharma)  Hydromorphone controlled-release | HCR | 24 | Capsule | 02125382 | 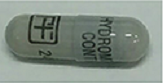 |
| Dilaudid (Purdue Pharma)  Hydromorphone immediate-release | HIR | 8 | Tablet | U20170104 | 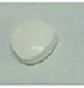 |
| Oxycontin  (Purdue Pharma)  Oxycodone controlled-release | OCR | 80 | Tablet | Lot# 10097054 | 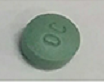 |
