# Supplementary material for: A controlled-release oral opioid supports S. aureus survival in injection drug preparation equipment and may increase bacteremia and endocarditis risk
Source: PLoS One. 2019 Aug 9;14(8):e0219777. doi: 10.1371/journal.pone.0219777 (PMC6688832; doi:10.1371/journal.pone.0219777)
Supplement: S2 Table — (DOCX) [file pone.0219777.s003.docx]

S2 Table. Clinical bacterial isolates from active PWID that were used in this study

| Strain | Species | Features | Source |
| --- | --- | --- | --- |
| LHSC-SAB-0001 | *S. aureus* | MSSA | Patient blood culture, PWID  SAB collection, McCormick Laboratory Collection |
| LHSC-SAB-0129 | *S. aureus* | MRSA | Patient blood culture, PWID  SAB collection, McCormick Laboratory Collection |
| IVDU-5 | *S. pyogenes* | Emm81 | Clinical sample from PWID with invasive infection, this study |
